# Supplementary figures and images for: Bulk and single-cell RNA sequencing identify prognostic signatures related to FGFBP2+ NK cell in hepatocellular carcinoma
Source: PeerJ. 2025 May 20;13:e19337. doi: 10.7717/peerj.19337 (PMC12101446; doi:10.7717/peerj.19337)

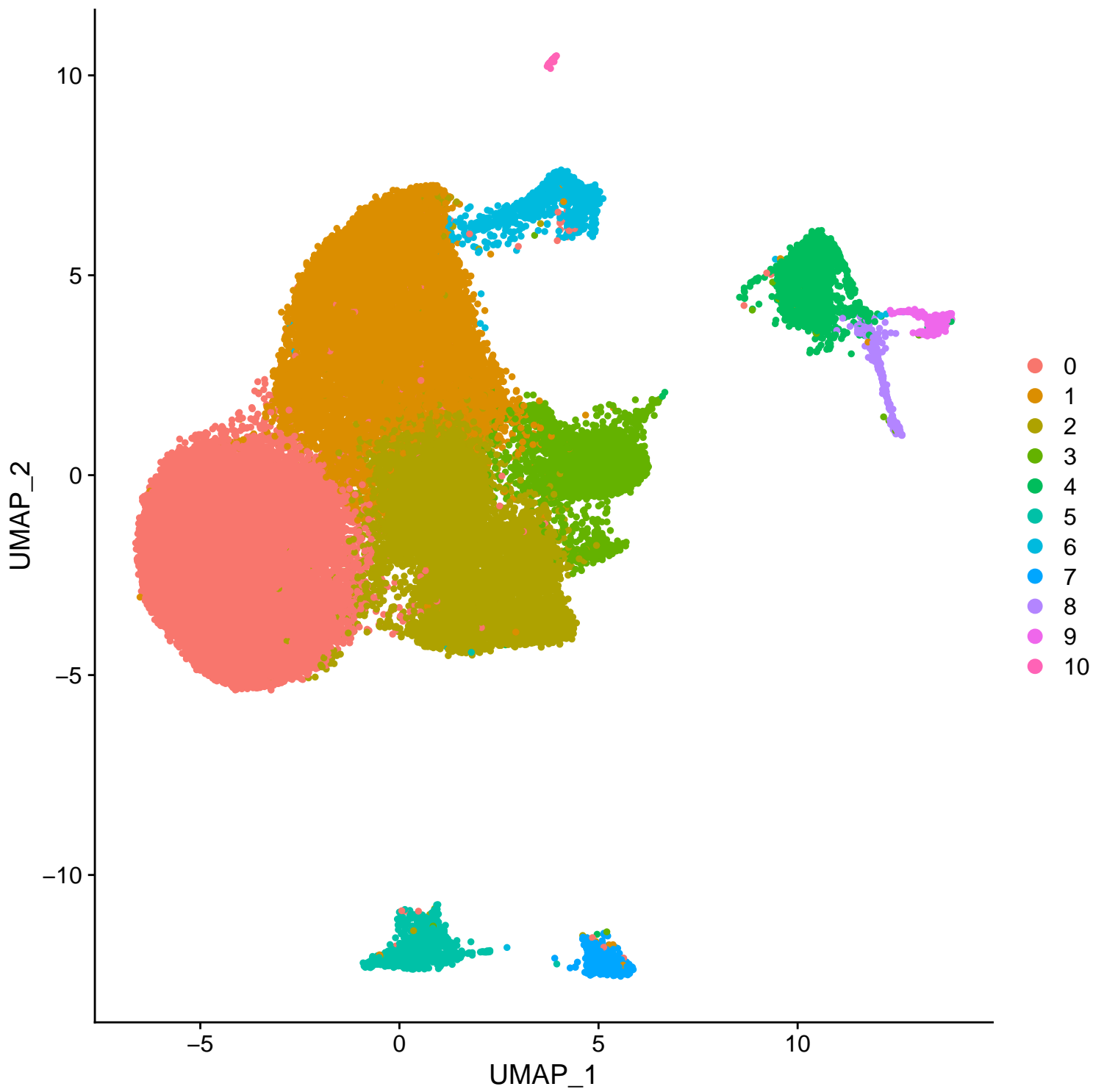

Supplement: Supplemental Information 1 [file peerj-13-19337-s001.pdf]

Risk 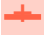 Score=high 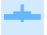 Score=low

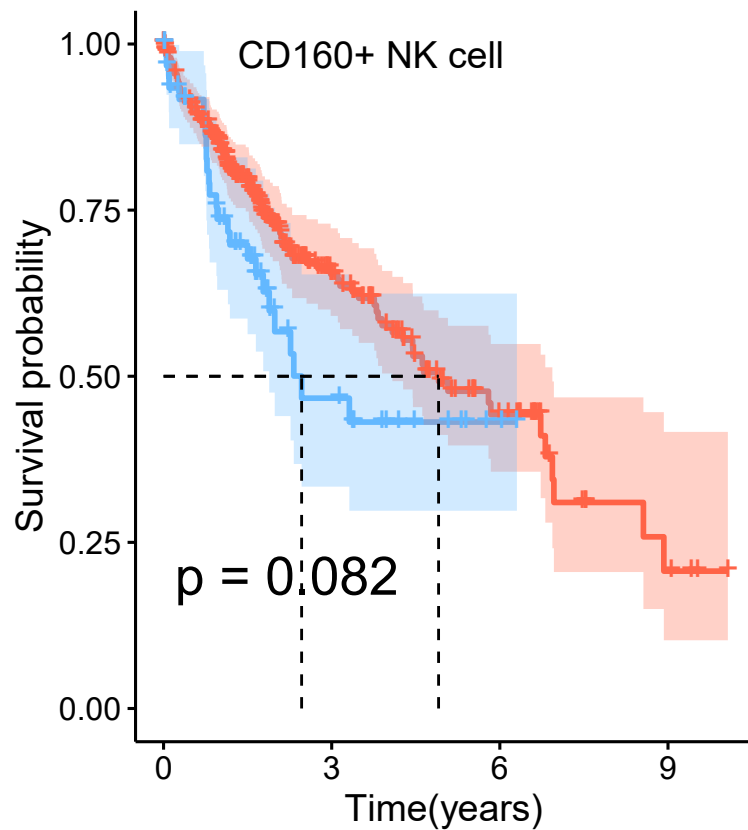

Risk 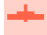 Score=high 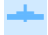 Score=low

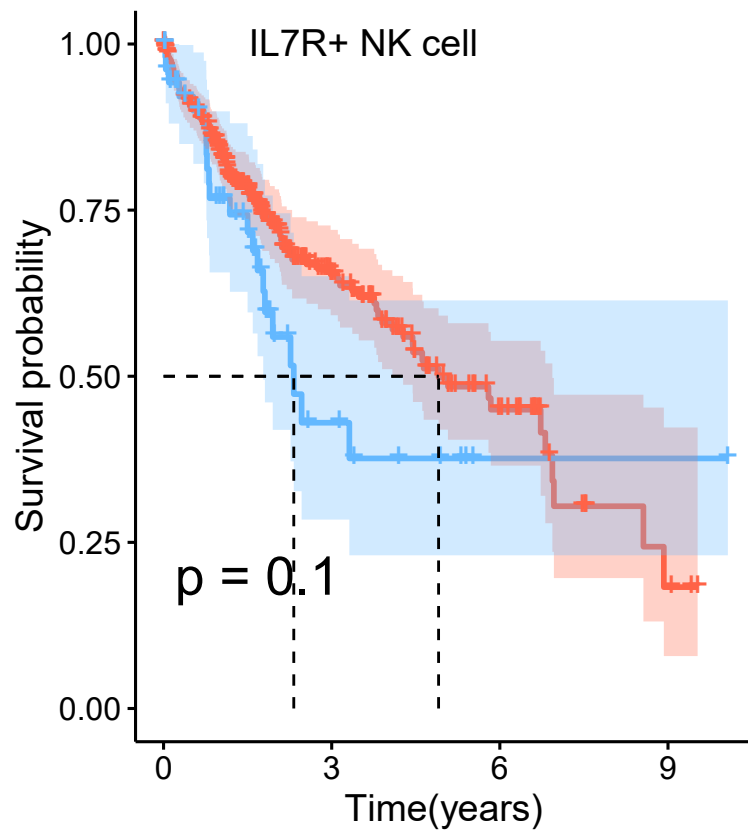

Supplement: Supplemental Information 2 [file peerj-13-19337-s002.pdf]

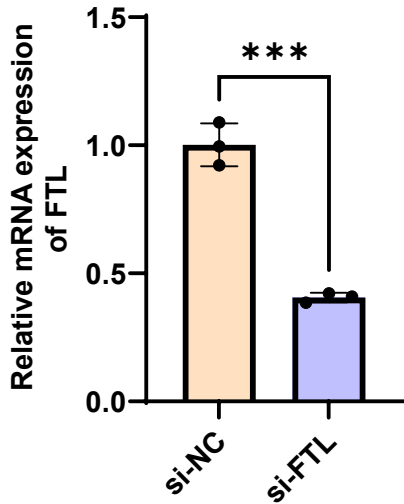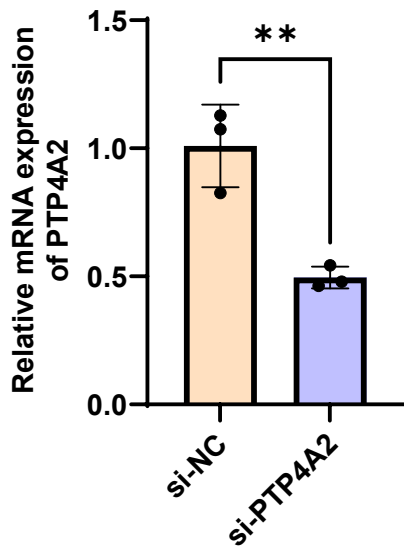

Supplement: Supplemental Information 3 [file peerj-13-19337-s003.pdf]
