# Supplementary material for: Bulk and single-cell RNA sequencing identify prognostic signatures related to FGFBP2+ NK cell in hepatocellular carcinoma
Source: PeerJ. 2025 May 20;13:e19337. doi: 10.7717/peerj.19337 (PMC12101446; doi:10.7717/peerj.19337)
Supplement: Supplemental Information 4 [file peerj-13-19337-s004.docx]

**Supplementary Table 1. Primer sequences used in qRT‑PCR**

| Gene | Primers (5’-3’) |
| --- | --- |
| *GZMH* | Forward: CTGGCTGGGGTTATGTCTCAA  Reverse: GGCTACGTCCTTACACACGAG |
| *KLF2* | Forward: TTCGGTCTCTTCGACGACG  Reverse: TGCGAACTCTTGGTGTAGGTC |
| *FTL* | Forward: CTCTCTCTGGGCTTCTATTT  Reverse: TTGATAAGCTTCACTTCCTC |
| *PTP4A2* | Forward: GAAGTATGGAGTGACGACTT  Reverse: ATCTTCGTACTTCATTCCAC |
| *UBE2F* | Forward: GGTTTCTGTGAGAGACAAAT  Reverse: GCATAACGTTTGATGTAGTC |
| *CDKN2D* | Forward: AGTCCAGTCCATGACGCAG  Reverse: ATCAGGCACGTTGACATCAGC |
| *RGS2* | Forward: CCGTTTGAGCTACTTCTTAC  Reverse: AAGAGTTGTTCTCCATCAAG |
| *AHSA1* | Forward: GTGTGAGGTGACGGAAGTGAG  Reverse: ACCTGTCCAGTTTAGTTTGACG |
| *GAPDH* | Forward: GGAGCGAGATCCCTCCAAAAT  Reverse: GGCTGTTGTCATACTTCTCATGG |
